# *Platycodon grandiflorum*, as a medicinal and food homologous plant: a comprehensive review of anti-tumor components, mechanisms, modern applications, and preventive healthcare

**DOI:** 10.3389/fnut.2025.1674705

**Published:** 2025-10-27

**Authors:** Wenjing Liu, Shumin Jia, Xiaoyan Ma, Doudou Lu, Yuhua Du, Ziying Zhou, Ling Yuan, Rong Yu, Yi Nan

**Affiliations:** ^1^The First Hospital of Hunan University of Chinese Medicine, Hunan University of Chinese Medicine, Changsha, Hunan, China; ^2^Key Laboratory of Dryness Syndrome in Chinese Medicine, Ministry of Education, Ningxia Medical University, Yinchuan, Ningxia, China; ^3^Department of Chinese Medical Gastrointestinal, The Affiliated TCM Hospital of Ningxia Medical University, Wuzhong, China; ^4^Department of Clinical Medicine, Ningxia Medical University, Yinchuan, Ningxia, China; ^5^Department of Pharmacy, Ningxia Medical University, Yinchuan, Ningxia, China; ^6^Department of Pharmacy, General Hospital of Ningxia Medical University, Yinchuan, China

**Keywords:** *Platycodon grandiflorum*, tumor, homology of medicine and food, anti-tumor active ingredients, anti-tumor mechanisms, novel drug delivery systems, preventive healthcare

## Abstract

*Platycodon grandiflorum* (Jacq.) A. D C. (*P. grandiflorum*), as a medicinal and edible plant, is widely distributed in China. In terms of Chinese medical theories, Traditional Chinese Medicine (TCM) believes that the root of *P. grandiflorum* has the functions of relieving sore throat, eliminating phlegm, and draining pus, and is widely used in lung diseases and respiratory system disorders. Modern pharmacological studies have shown that *Platycodon grandiflorum* saponin, flavonoids, polysaccharides, and phenolic acids are the main active components of *P. grandiflorum*, which have significant antitussive, expectorant, anti-tumor, anti-oxidation and hypoglycemic effects. In recent years, an increasing number of research evidence has demonstrated the anticancer potential of *P. grandiflorum*. In this study, we firstly identified the anti-tumor active components of *P. grandiflorum* (platycodin D, *P. grandiflorum* polysaccharides, lobetyolin, luteolin, and apigenin) as well as their mechanism of action in inhibiting cancer cell proliferation (inducing apoptosis, blocking the cell cycle and inhibiting tumor metastasis). Additionally, we investigated the detoxification and synergy-enhancing effects of these active components when combined with chemotherapeutic drugs, as well as the improvement of their drug delivery and the bioavailability through nano-delivery systems, which hold great anticancer potential for clinical application. However, research on the anti-tumor effects of *P. grandiflorum* extract and its active components still lacks large-scale clinical trials. Finally, we further summarized the applications of *P. grandiflorum* in preventive healthcare, emphasizing its dual value in the pharmaceutical and food industries. This summary provides a scientific basis for further research on its anti-tumor properties and highlights its potential development in modern preventive healthcare.

## Graphical abstract

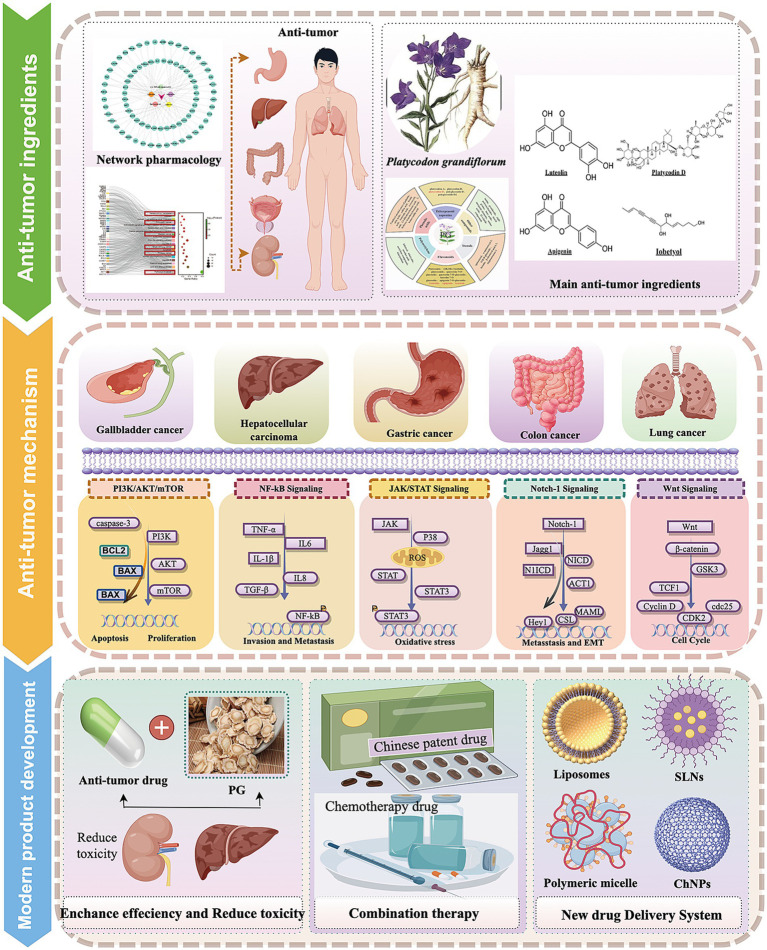

## 1 Introduction

Malignant tumors are a class of diseases with complex pathogenesis, poor prognosis, and high morbidity and mortality rates. According to World Health Organization (WHO) reports, 17.5 million cancer patients deaths and 27 million new cancer patients will happen annually by 2050 (1). Nowadays, traditional treatment methods still form the backbone of clinical tumor treatment, including surgery, radiotherapy, and chemotherapy. However, these methods often cannot avoid affecting normal tissues-thus having the characteristic of “non-specificity” (2). In addition, targeted therapy for tumors is a precise treatment method that selectively targets specific receptors on tumor cells. However, targeted therapy also encounters challenges, including tumor cell resistance, high costs, a diversity of therapeutic targets, and potential side effects (3). Therefore, continued research and development of new treatment methods are crucial to improve treatment effectiveness and safety.

Traditional Chinese medicine (TCM), as one of the effective means of prevention and treatment of malignant tumors, has achieved significant efficacy in tumor treatment. Active natural compounds derived from traditional Chinese herbs play a crucial role in cancer treatment by producing anti-cancer effects, effectively improving clinical effects, and reducing the toxic side effects associated with chemotherapy and radiation therapy. According to the literature, natural products have a more diverse structure and can effectively exert specific biological functions. For example, they mainly regulate the body’s internal environment to inhibit tumor growth, reduce tumor metastasis, and modulate immune responses (4). Statistics show that a significant proportion of FDA-approved anti-cancer drugs are derived from natural plants. This makes natural compounds promising and practical alternatives for cost-effective, highly effective, and safer cancer treatments and prevention. However, currently only 2.5–5 million plant species have been thoroughly studied, which means there is still enormous potential to be explored in the development of anti-cancer therapies based on natural plants.

*Platycodon grandiflorum* (Jacq.) A. D C. (*P. grandiflorum*), known as “Jiegeng” or “Lingdanghua” (in China), “Doraji” (in Korea) and “Kikyo” (in Japan), is a dried root of the *Platycodon* genus, which attributes to the Campanulaceae family (5). The Campanulaceae family has about 60–70 genera, and *P. grandiflorum* is the only genus in this family (6). *P. grandiflorum* is primarily found in the northeastern, northern, and central regions of China, along with Korea, Japan, the Russian Far East, and southeastern Siberia (7). According to a record of TCM dated 2000 years ago, *P. grandiflorum* was first mentioned in “*Shennong Bencao Jing*” (a famous monograph of TCM). The ancient book stated that *P. grandiflorum* had a slightly warm and pungent taste, and it was used to treat chest and hypochondriac pain, fullness in the abdomen, faint intestinal sounds, and palpitations caused by fear. Subsequently, throughout different historical periods, medical scholars have consistently described the taste and efficacy of *P. grandiflorum*. *“Mingyi Bie Lu,” “Xinxiu Bencao,” “Kaibao Bencao,” “Tangye Bencao,” “Jingyue Quanshu,” and “Bencao Gangmu”* all described *P. grandiflorum* having a bitter and slightly warm taste, which can relieve sore throat and help to evacuate pus. Since the Qing Dynasty, people have commonly used *P. grandiflorum* to make pickled vegetables in northeastern of China. Therefore, *P. grandiflorum* is a medicinal and edible species, which has great potential in the development and application of medicine, health care products, dietary supplements and food (8).

Currently, the rhizome of *P. grandiflorum* has been widely used in TCM with remarkable ability to promote lung function, ease sore throat, eliminate phlegm and pus, as determined by the Committee for the Pharmacopoeia of PR China (2020). Modern pharmacological studies have shown that the medicinal component of *P. grandiflorum* is its root, which has various pharmacological effects such as cough suppression (9), immune stimulation (10), anti-inflammatory activity (11), anti-oxidant activity, and anti-tumor activity (12). According to the literature, an increasing number of research evidence has demonstrated the anticancer potential of *P. grandiflorum*. However, there is a notable absence of comprehensive reports detailing the anti-tumor effects of *P. grandiflorum*. It has been shown that the active ingredients of *P. grandiflorum*, such as saponins, flavonoids and polysaccharides play important roles in inhibiting tumor cell proliferation, inducing cell apoptosis and cell autophagy, inhibiting the invasion and migration of tumor cells, regulating immune function, inhibiting tumor angiogenesis (13).

Before writing this review, we reviewed the literature search in PubMed,^1^ Google Scholar,^2^ ScienceDirect,^3^ Web of Science,^4^ and other databases using the keywords of “*Platycodon grandiflorum*” AND (“anti-tumor” OR “anti-cancer”) and *“Platycodin D*” AND (“anti-tumor” OR “anti-cancer”) from the past 5 years. Fortunately, two reviews attracted our attention, primarily focusing on summarizing the botanical description, distribution, traditional uses, chemical composition, and pharmacological effects of *P. grandiflorum* (5, 14). However, due to the over-comprehensive describe the *P. grandiflorum* efficacy, only a brief summary on the anti-tumor potential of *P. grandiflorum* provided without a comprehensive discussion on the antitumor mechanism. Consequently, to address this research gap, we conducted a comprehensive and systematic review in the present study. The purpose of this review is to systematically clarify the anti-tumor potential of *P. grandiflorum*, including its anti-tumor components (platycodin D, *P. grandiflorum* polysaccharides, luteolin, apigenin, and lobetyolin), mechanisms, application prospects, and preventive healthcare, with the following research objectives and implementation processes, shown as in graphical abstract.

## 2 Network pharmacological analysis of *P. grandiflorum*

**Figure 1:**
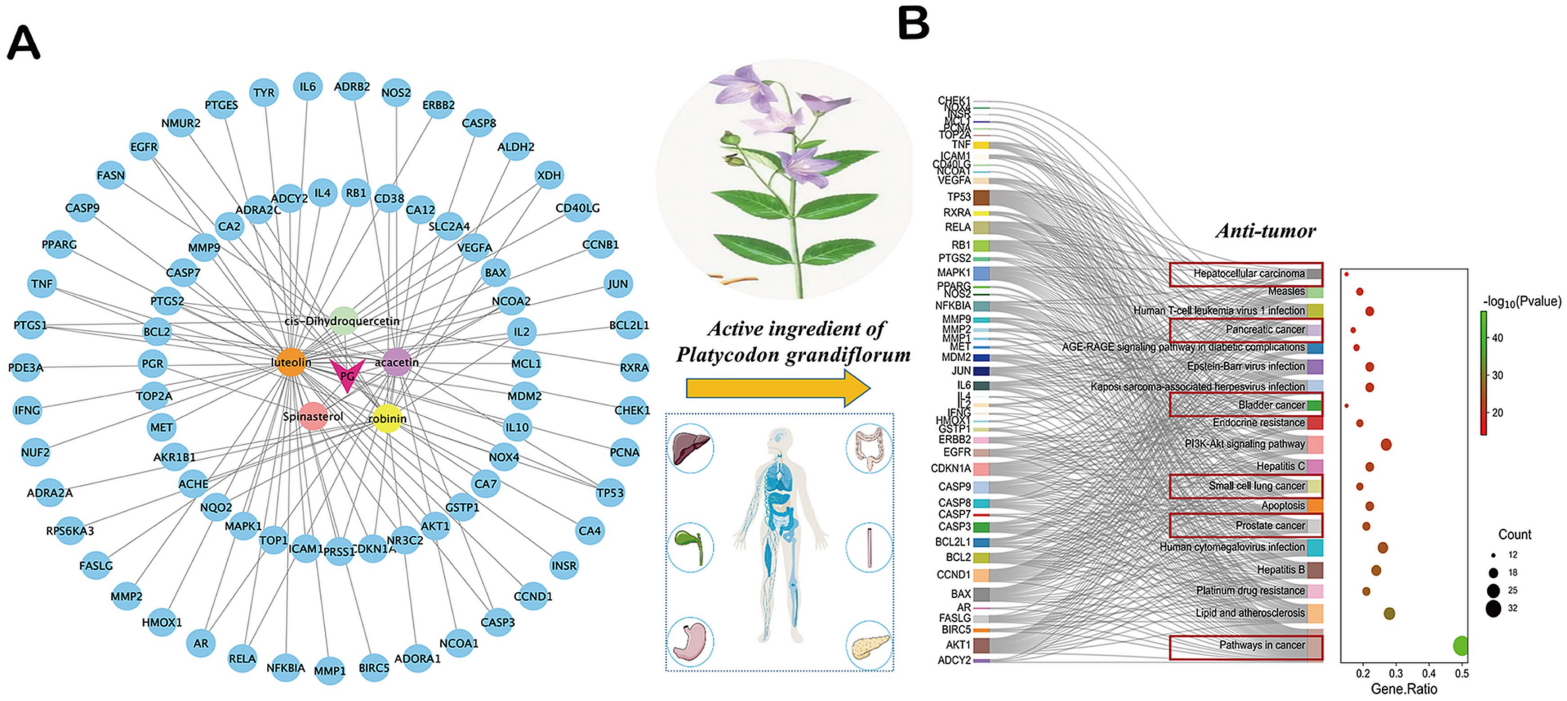
Network pharmacological analysis of related active ingredients, targets, and pathways in *P. grandiflorus*. **(A)** Network diagram of active ingredients and targets in *P. grandiflorus*. **(B)** The KEGG pathway of active ingredients from *P. grandiflorus* anti-tumors. The red box represents the type of tumor treatment.

The active ingredients of *P. grandiflorum* were analyzed by TCMSP database^5^ (accessed May 10, 2025) using the screening criteria of Drug-Like (DL) ≥ 0.18 and Oral Bioavailability (OB) ≥ 0.3. The active components were obtained and their corresponding ID, Molecule Name, OB and DL values were displayed in Supplementary Table 1. The targets of *P. grandiflorum*’s active ingredients were predicted by TCMSP database, and the targets were corrected by UniProt database^6^ (accessed May 12, 2025) and converted to gene targets. Additionally, SwissTargetPrediction database^7^ (accessed May 12, 2025) was incorporated to further supplement the active ingredients of *P. grandiflorum* and their corresponding gene targets. We performed Kyoto Encyclopedia of Genes and Genomes (KEGG) analysis through the Metascape database^8^ (accessed May 12, 2025), the pathway information was obtained and their corresponding Description, Gene Ratio, gene ID, and Count were displayed in Supplementary Table 2. Next, we visualized the result through the Wei Shengxin platform.^9^ Based on the screening criteria, a total of five active ingredients and 95 targets of *P. grandiflorum* were obtained in the TCMSP database. After importing into Cytoscape3.8 (The Cytoscape Consortium, United States), the “target-ingredient” network was constructed in Figure 1A. Then, these drug targets were imported into the Metascape database. The results indicated that among the top 20 signaling pathways enriched in *P. grandiflorum* drug targets, six pathways were primarily associated with cancer, including hepatocellular cancer, pancreatic cancer, bladder cancer, small cell lung cancer, prostate cancer, pathways in cancer, etc. (Figure 1B). Therefore, based on the network pharmacology results, we have reason to believe that *P. grandiflorum* has widely anti-tumor effects.

## 3 Main active ingredients of *P. grandiflorum*

**Figure 2:**
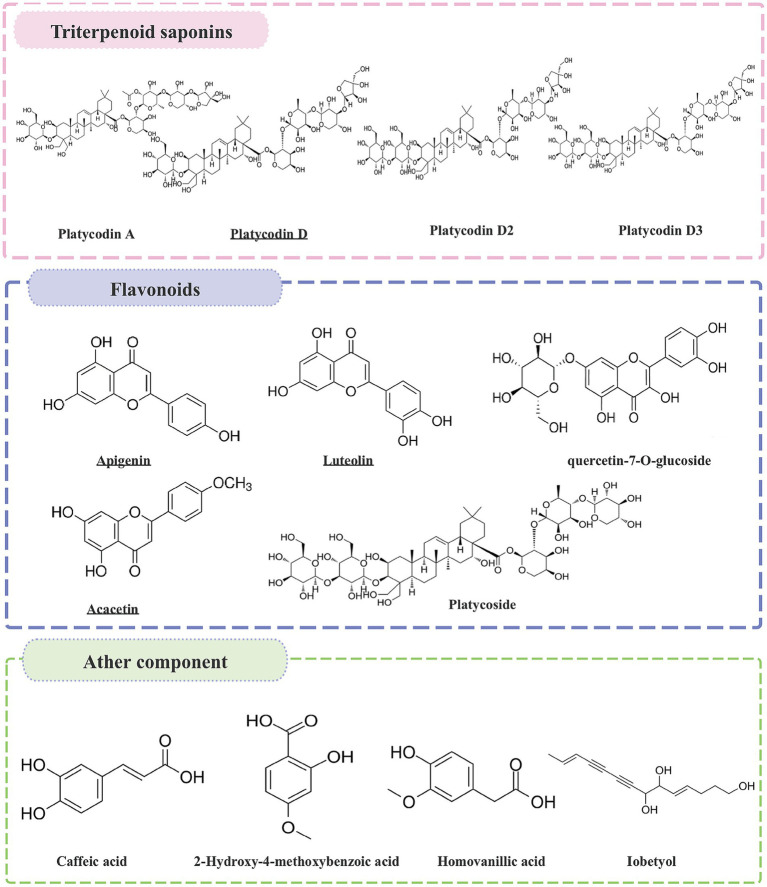
The chemical structures of the active ingredients in *P. grandiflorus* with potential medicinal functions.

The roots of *P. grandiflorum* contain high levels of carbohydrates (90%), protein (2.4%), fat (0.1%), ash (1.5%), and approximately 2% triterpene saponins (15). Over the past several decades, at least 100 compounds have been isolated from *P. grandiflorum*, including steroidal saponins, flavonoids, polysaccharide, phenolic acids, polyacetylenes, sterols, fatty oils, fatty acids, various amino acids, and vitamins, etc. (16, 17). The variety of natural chemical components makes the pharmacological applications of *P. grandiflorum* very broad. The *P. grandiflorum* main active components (Table 1) and chemical structures with potential medicinal functions (Figure 2).

**Table 1:** Main active ingredients and efficacy of *P. grandiflorum.*

	Ingredient	Efficacy	References
Triterpenoid saponins	Platycodin A, platycodin B, platycodin C, platycodin D, platycodin D2, deapioplatycodin D, platycodin D3, etc.	Antitussive and expectorant, antioxidant, anti-inflammatory, anti-tumor, regulating metabolism, liver protection, immune regulation	(19, 20, 134)
Flavonoids	Apigenin, luteolin, acacetin, tangerine, quercetin-7-O-glucoside, quercetin-7-O-rutin, delphinidin-3-rutin-7-glucoside, etc.	Antioxidant, anti-tumor, antimicrobial, anti-inflammatory, anti-apoptotic, anti-allergic, anti-diabetic, cardioprotective, and neuroprotective	(21, 22)
Polysaccharides	*α*-spinasteryl-3-O-*β*-D-glucoside, L-Arabinose, D-Glucuronic acid, L-Rhamnose, D-Galactose, D-Mannose, etc.	Immune regulation, anti-tumor, hypoglycemic, anti-aging and anti-viral effects	(23–25)
Other components	Phenolic acids, lobetyolin, polyacetylene, sterols, amino acids, caffeic acid, 3,4-dimethoxycinnamic acid, ferulic acid, isoferulic acid, m-coumaric acid, p-coumaric acid, p-hydroxybenzoic acid ɑ-Phthalic acid, 2,3-dihydroxybenzoic acid, 2-hydroxy-4-methoxybenzoic acid, homovanillic acid, and chlorogenic acid. Fatty acids, etc.	Antioxidant, blood lipid regulation, anti-tumor, anti-inflammatory, immune regulation, neuroprotective properties	(28, 135)

First of all, triterpene saponins are a class of compounds found in many plants. Platycodin refers to a variety of purified cholane-type triterpene saponins with diverse pharmacological activities present in the roots of *P. grandiflorum* (18). There are numerous types of Platycodin, including platycodin A, platycodin B, platycodin D, Polygala D, Polygala D2, etc. (19). Among all the saponin components, platycodin D is the first saponin isolated from *P. grandiflorum*, and is recognized as one of the most potent pharmacological components (15). It has been reported to have antitussive, anti-inflammatory, anti-tumor, regulating metabolism effects (20). Subsequently, Flavonoids are primarily found in the aboveground parts of *P. grandiflorum* and can be classified into flavonoids, dihydroflavonoids, and flavonoid glycosides. To date, more than 10 flavonoid components have been isolated from the aboveground parts (21), including apigenin, luteolin, acacetin, tangerine, quercetin-7-O-glucoside, quercetin-7-O-rutin, delphinidin-3-rutin-7-glucoside and others. Among these components, apigenin and luteolin are the main constituents (22). According to relevant studies, luteolin glycosidic acid and luteolin 7-glucoside exhibit antioxidant, anti-tumor, antimicrobial, anti-inflammatory, anti-apoptotic, anti-allergic, and neuroprotective properties (23). *P. grandiflorum* polysaccharide is an important active ingredient in *P. grandiflorum* (24). Polysaccharide is composed of fructose, mannose, xylose, and arabinose; mannose is the main sugar component (25). Currently, polysaccharides with known structures have been identified, such as the eustoma polysaccharides GF2 ~ GF9. They have significant pharmacological effects in enhancing immune function, anti-tumor, hypoglycemic, anti-aging and anti-viral effects, which are well documented in the China Pharmacopoeia (26). Park et al. (27) found that *P. grandiflorum* polysaccharide can effectively inhibit the autophagic damage induced by chromium (VI) [Cr(VI)] to the mitochondria of DF-1 cells. In addition to saponins, flavonoids, and polysaccharides, *P. grandiflorum* also contains active ingredients such as phenolic acids, lobetyolin, polyacetylene, sterols, and amino acids. Lee et al. (28) isolated two phenolic compounds from the roots of *P. grandiflorum*, namely coniferyl palmitate and coniferyl oleate.

## 4 Extraction process and detection method of antitumor components from *P. grandiflorum*

In recent years, the extraction of active components from natural plants using safe, eco-friendly, and efficient methods has gained significant popularity. The extraction methods of natural compounds mainly include water extraction, alcohol extraction, cold immersion extraction, ultrasonic extraction and supercritical extraction, among which organic solvent extraction method is the most widely used method. In addition, many determination techniques have been developed for plant extracts (29). Among these determination methods, high-performance liquid chromatography (HPLC) is the most widely employed, and it is characterized by high sensitivity, excellent repeatability, and straightforward standardization (30).

In this review, we focused primarily on the anti-tumor active components and extraction methods of *P. grandifloram. P. grandiflorum*, as a plant with both medicinal and edible properties, contains various anti-tumor active components-including platycodin D (31), luteolin, apigenin (32, 33), polysaccharides (34) and lobetyolin (35). Currently, the extraction methods and processes for these components have been extensively studied (36, 37). Jingyin et al. (38) determined that the content of *platycodin D* has been recognized as the major active compound in *P. grandiflorum* roots than that of leaves. Kwon et al. (39, 40) used HPLC-ELSD to determine that the content of platycodin D in *P. grandiflorum* root varies depending on three processing methods: alcohol extraction, water extraction, and ultrasonic-assisted extraction. In addition, luteolin and apigenin, as flavonoids, are mainly found in the above-ground parts of the *P. grandiflorum.* Zhou et al. (41) used ultrasonic extraction and HPLC simultaneous determined the contents of luteolin and apigenin in *P. grandiflorum.*

Currently, the extraction of polysaccharides has attracted significant attention. Zhang et al. (42) used water reflux extraction and ultrasonic extraction method to extract polysaccharides of *P. grandiflorum,* among them, the water reflux extraction efficiency is as high as 44%. In addition, there are other methods for polysaccharides. Zhang et al. (43) determined the content of polysaccharides in the roots of *P. grandiflorum* from China’s three major production areas via the phenol-concentrated sulfuric acid method, and reported that the content ranged from 18.1 to 23.1% (44). Meanwhile, Yin et al. (45) used ultrasonic extraction and HPLC determined the contents of platycodin D and lobetyolin from *P. grandiflorum* (The content of platycodin D was 5.12 ± 0.13 mg/g, and the yield of lobetyolin was 0.14 ± 0.01 mg/g). Since lobetyolin is mainly found in the root of the *P. grandiflorum*, its extraction usually uses the root as the raw material. Chen et al. (46) employed accelerated solvent extraction methods and used HPLC to detect content level of lobetyolin. In conclusion, we summarized the extraction sites, extraction processes and detection methods of these anti-tumor components in *P. grandiflorum*, as shown in Table 2.

**Table 2:** Extraction process and detection method of antitumor components from *P. grandiflorum.*

Target component	Extraction sites	Extraction process	Detection method	Content	References
Platycodin D	Root (skin/cortex)	Alcohol extraction, water extraction, and ultrasonic-assisted extraction	HPLC-ELSD	5.12 ± 0.13 mg/g	(40, 136, 137)
Luteolin	Root/Leaves/Flowers	Alcohol extraction, water extraction, and ultrasonic-assisted extraction	HPLC	0.224 mg/g	(41)
Apigenin	Root/Leaves/Flowers/seed	Alcohol extraction, water extraction, and ultrasonic-assisted extraction	HPLC-UV	0.42 mg/g	(138)
Polysaccharide	Root (pith)	Water reflux extraction method, microwave-assisted extraction method and ultrasonic extraction method, phenol-concentrated sulfuric acid method	HPLC	18.1–23.1%	(37, 139)
Lobetyolin	Root	Accelerated Solvent Extraction Method (ASE), ultrasonic-assisted extraction	HPLC	0.14 ± 0.01 mg/g	(46, 140)

## 5 Anti-cancer effects and underlying mechanisms

In the anti-cancer research, Platycodin D is the main active component of *P. grandiflorum*, which has been the most extensively studied (47); followed by flavonoids and polysaccharides, which have also received significant research attention in anti-cancer studies. In addition, the network pharmacological analysis and literature retrieval of *P. grandiflorum* extracts and active components have been demonstrated which can inhibit multiple types of cancer (Colon cancer, gallbladder cancer, stomach cancer, hepatoma carcinoma cell lung cancer, etc.) and have anti-tumor activity by inhibiting several signaling pathways, including PI3K/AKT/mTOR, MAPK, JNK/STAT3, Wnt, Notch-1, and NF-κB signaling pathways, etc. Further research findings have indicated that *P. grandiflorum* has promising therapeutical potential for cancer treatment. It inhibits cancer cell proliferation, induces cancer cell apoptosis, suppresses cancer cell migration and invasion, inhibits angiogenesis, and regulates autophagy, etc. (Table 3).

**Table 3:** Anticancer components and mechanism of *P. grandiflorum.*

Ingredient	Cancer type	Experimental Model	Mechanism	Phenotype	References
Platycodin D	Colorectal cancer	KRAS mutant human colon cancer cell line HCT116, LoVo	Reduced migration and invasion; Inhibited proliferation; Enhanced apoptosis; Inhibition of p-PI3K and p-AKT.	Proliferation, apoptosis, migration, invasion	(52)
	Hepatocellular carcinoma	Human Hepatocellular carcinoma HA22T and HDACi-R cells	Inhibited proliferation; Induced apoptosis; Reversed HDACi liver cancer while inhibiting ERK1/2 mediated cofilin-1 phosphorylation	Proliferation and apoptosis	(141)
		Human Hepatocellular carcinoma BEL-7402 cells	Inhibited proliferation; Induced apoptosis; Downregulation of Bcl-2 expression level, upregulation of Bax/Bcl-2 ratio, and cleavage of PARP and Caspase-3 levels.	Proliferation, apoptosis, autophagy	(91)
	Non-small cell lung cancer	Human Non-small cell carcinoma line A549 and H46 cells	Inhibited cell proliferation; Induced apoptosis and upregulated the cleavage of apoptotic proteins caspase-3, caspase-9, and PARP; Induced G0/G1 phase arrest in both cell lines; inhibited the expression of TIMP-1, CDK2, cyclin A, and cyclin E; By inhibiting GSK3β, p-AKT and the expression of p-PI3K blocked the PI3K/AKT signaling pathway.	Proliferation and apoptosis	(68)
	Head and neck cancer	Human oral squamous cell carcinoma OSCC cells	Inhibited cell viability; induced cell apoptosis, and inhibited cell invasion through NF- κ B signaling pathway	Proliferation, apoptosis and migration	(76)
Apigenin	Prostatic cancer	Prostate cancer cell lines LNCaP and PC-3	Induced G2/M blocked to inhibit the proliferation.	Proliferation	(142)
	Non-small cell lung cancer	Human Non-small cell carcinoma lines A549, H129 cells	Inhibition of cell proliferation and induced apoptosis; Blocked G2/M cell cycle; Improved mitochondrial dysfunction; Increased oxidative stress, and activated Apoptosis signaling pathways.	Proliferation and apoptosis	(143)
	Hepatocellular carcinoma	Hep G2 cells of human liver cancer	Induced apoptosis; Upregulation of PTEN protein expression; Inhibited p-AKT and downregulated p-Bad.	Apoptosis	(70)
Luteolin	Gastric cancer	Human Gastric cancer cell lines NCI-N87 and MKN28	Inhibited cell proliferation, migration, and invasion; Promoted cell apoptosis; Reversing EMT to inhibit Notch1 signaling pathway.	Proliferation, migration, invasion, apoptosis	(80)
	Hepatocellular carcinoma	Human Liver cancer cell line SMMC-7721	Reduced cell vitality; Induced G0/G1 phase arrest; Promoted cell apoptosis.	Proliferation and apoptosis	(92)
	Colorectal cancer	Colon cancer cell lines SW620 and SW480 cells	Inhibited cell proliferation and migration/invasion by inhibiting the IL-6/STAT3 signaling pathway.	Proliferation, migration, invasion	(81)
	Colorectal cancer	Human ovarian cancer cell line HGSOC cells	Inhibited HGSOC cell proliferation and activated the PI3K/AKT signaling pathway	Proliferation and apoptosis	(144)
		Xenograft tumor model	Inhibited cell viability, colony formation, migration, invasion, and promoting apoptosis of lung cancer cells; By adjusting the circle_0000190/miR-130a-3p blocked the growth, metastasis, and Notch-1 signaling pathway.	Proliferation, apoptosis and migration	(145)
*P. grandiflorum* polysaccharides	Cervical cancer	HeLa cancer cells	Suppressed the growth of U-14 transplanted tumors by up-regulating the p19ARF and Bcl-2 and down-regulating the expression of mutant p53, thereby inducing apoptosis in cancer cells.	Proliferation, apoptosis	(146)
	Lymphoma	S180 tumor-bearing mice	Decreased protein expression levels of TLR4, MyD88, and NF-κB	Proliferation, invasion, migration	(73)
	Cervical cancer	U14 cervical cancer bearing mice	Suppressed the growth of U-14 transplanted tumors by up-regulating the p19ARF and Bcl-2 and down-regulating the expression of mutant p53	Proliferation, apoptosis	(72)
Lobetyolin	Gastric cancer	Human Gastric cancer cell lines MKN-45 and MKN-28	Down-regulation of ASCT2 inhibited the protein levels of the AKT/GSK3β/c-Myc pathway, thereby inhibiting cell proliferation and inducing cell apoptosis.	Proliferation, apoptosis	(147)
	Colon cancer	Human Colon cancer cell HCT-116 and NCM460	Regulated ASCT2 through the p53-dependent pathway, activate caspase-3/7 and PARP cleavage, and induce apoptosis	Proliferation, apoptosis	(35)
	Non-small cell lung cancer	Balc/b nude mice model of lung cancer	Reduced the expression of vimentin and MMP9 in the tumor tissues, while increased the expression of E-cadherin. The mechanism might be related to epithelial-mesenchymal transition (EMT).	Invasion, migration	(148)

### 5.1 Inhibiting cell proliferation and blocking cell cycle

Excessive cell proliferation is one of the key indicators of cancer development, and cancer cells possess the capability to evade apoptosis through the activation of survival support signals, which stands as a significant characteristic. The PI3K/AKT signaling pathway is commonly overexpressed in a variety of human cancers (48). The c-Myc transcription factors play an essential role in normal, untransformed cells, regulating proliferation, differentiation, cell growth, and apoptosis (49). Cancer cells often escape terminal differentiation by increasing the c-Myc expression, which is an effective inhibitor of differentiation in numerous cell lines (50). In gastric cancer, platycodin D exerts its anti-cancer effect by promoting c-Myc protein ubiquitination and degradation (51). Liu et al. (52) found that platycodin D can suppresses tumor cell growth *in vitro* and vivo by down-regulating the p-PI3K and p-AKT expression in HCT116 cells. Zhou et al. (53) found that the water extract of *P. grandiflorum* inhibits the PI3K/Akt signaling pathway, thereby reducing the apoptosis of lung cells and the infiltration of inflammatory cells in mice induced by lipopolysaccharide.

In addition, abnormal cell cycle progression is also one of the basic mechanisms of tumor proliferation (54). Cyclin B1 is a crucial constituent of the cyclin family, which can activate specific cyclin dependent kinases (CDKs) required for cell cycle progression (55). Activated Cyclin B1 can promote cells from the G2 phase to the M phase, and initiate mitotic progression (56). In the study, Lee et al. (57) found that apigenin can trigger G2/M cell cycle arrest, inhibit the expression of Cyclin B1, Cdc2, and Cdc25c, thereby inhibiting the proliferation of HCT116 colon cancer cells. In their study, Ujiki et al. (58) discovered that the growth of pancreatic cancer cells is hindered by apigenin due to its ability to suppress the G2/M cell cycle and decreased the activity of CyclinB1-Cdc2. In ovarian cancer, luteolin induced cell apoptosis and arrested cell cycle in G2/M by reducing the expression of VRK1 and activating the p53 signaling pathway, and inhibited HGSOC cell proliferation (59). P53 is a transcription factor responsible for inhibiting tumor growth. Its main anti-tumor mechanisms include inhibiting cell proliferation by blocking the cell cycle and promoting cell apoptosis (60). In the study of esophageal cancer, luteolin can inhibit the proliferation and growth of EC1, EC9706, KYSE30, and KYSE450 cells; Up-regulation of the expression of cell cycle inhibitory proteins p21 and p53 inhibits ESCC cells proliferation (61). He et al. (62) evaluated the tumor growth inhibitory effects of two *P. grandiflorum* polysaccharides (PRP1 and PRP2) isolated and purified from hot water extraction on liver cancer. PRP1 inhibited the proliferation of HepG2 cells by down-regulating miR-21 and activating PTEN. Cai et al. (63) extracted and purified polysaccharides from *P. grandiflorum* and conducted *in vitro* cell experiments. The results confirmed that *P. grandiflorum* polysaccharides promote apoptosis in liver cancer cells by enhancing the expression of LINC01554 (a long non-coding RNA), thereby inhibiting the progression of liver cancer.

### 5.2 Inducing tumor cell apoptosis

Apoptosis is a cell suicide plan executed by caspases, which is crucial for maintaining tissue homeostasis. Impaired apoptosis is now known to be a key step in cancer development. Whether a cell is alive or dead is largely determined by the anti-apoptotic modulators of the Bcl-2 family, and the release of cytochrome C into the cytoplasm is a crucial event in mitochondrial apoptosis, and it is regulated by the Bcl-2 protein family. Therefore, Bcl-2 plays a crucial role in intrinsic apoptosis by regulating mitochondrial membranes (64). In bladder cancer, platycodin D can increase the expression of Caspase-9, Caspase-8, Caspase-3 and p53 in tumor tissue, and promote cancer cell apoptosis by reducing the expression of Bcl-2 (64). Zeng et al. (65) demonstrated that platycodin D upregulated the expression of cytochrome C by regulating Bcl-2 in gallbladder cancer, further validating the endogenous cell apoptosis mechanism induced by platycodin D. In addition, the anti-apoptosis mechanism of platycodin D has been confirmed in many cancers, such as breast cancer (50), colon cancer (52), gallbladder cancer (66), prostate cancer (67) and non-small cell lung cancer (68). In gastric cancer, a research has shown that luteolin can suppress the proliferation of MKN45 cells, induce apoptosis, lower the mitochondrial membrane potential, and increase the expression of BAX, Caspase3, and cytochrome C, and reduce the expression of Bcl-2 and p-AKT (69). As is well known, triggering tumor cell apoptosis is related to various signaling pathways (PI3K/AKT/mTOR, MAPK, JAK/STAT3, and NF-κB, etc.). In liver cancer research, apigenin can induce the expression of PTEN protein in human liver cancer cells, though inhibiting PI3K/AKT signaling and reducing the level of phosphorylated Bad protein, thereby achieving the effect of inducing liver cancer cell apoptosis (70); apigenin has also been proven to have therapeutic effects on cancer cells. Apigenin induces autophagy by inhibiting the activity of key molecules p-AKT and p-mTOR in gastric cancer cells, thereby enhancing the apoptotic ability of gastric cancer cells (71).

Lu et al. (72) used the polysaccharides extracted from dried *P. grandiflorum* root to conduct *in vitro* studies. They found that the *P. grandiflorum* polysaccharides could significantly inhibit the growth of HeLa cancer cells. Moreover, they could also suppress the growth of U-14 transplanted tumors by up-regulating the p19ARF and Bcl-2 and down-regulating the expression of mutant p53, thereby inducing apoptosis in cancer cells. Wang et al. (73) administered S180 tumor-bearing mice with *P. grandiflorum* polysaccharides by gavage. They found that the lymphocyte proliferation index significantly increased, while the protein expression levels of TLR4, MyD88, and NF-*κ*B significantly decreased. This might be related to the inhibition of tumor growth and the improvement of the body’s immune function.

### 5.3 Inhibiting tumor cell migration and invasion

Tumor invasion and migration is a complex, multi-step biochemical process that is considered to be one of the hallmarks of cancer biology. The degradation of the basement membrane and remodeling of the ECM are hallmarks of tumor invasion and metastasis (74). Abnormal expression of matrix metalloproteinases (MMPs), particularly MMP-2 and MMP-9, has been observed in tumors, and their involvement in tumor invasion and metastasis is widely recognized. Lee et al. (75) found that the aqueous extract of *P. grandiflorum* root inhibits tumor invasion and metastasis. Zhang et al. (76) found that platycodin D hindered NF-κ B regulates the activity of downstream genes of MMP-2 and MMP-9, thereby hindering the migration and invasion of oral squamous cell carcinoma cells. This phenomenon has also been confirmed in gallbladder cancer (66) and prostate cancer (77). In the study of liver cancer, platycodin D effectively hindered the growth of HePG2 and Hep3B cells by significantly suppressing the development of HepG2 cell colonies, and triggering cellular apoptosis, and inhibited TPA induced cell migration and invasion (78). Huang et al. (79) found that luteolin can inhibit the expression of EMT and MMP2, MMP7 and MMP9 in a dose-dependent manner, and inhibit the invasive ability of PANC-1 and SW1990 pancreatic cancer cells. In gastric cancer research, Zhang et al. (80) found that luteolin can significantly suppress the migration and invasion of NCI-N87 and MKN28 cell through the Notch1 pathway, induce cell apoptosis, and reverse the occurrence of EMT. In colorectal cancer, luteolin hindered migration and invasion of SW620 and SW480 cancer cells through inhibiting the STAT3 pathway (81). In non-small cell lung cancer, luteolin inhibited the growth of H460 cells by EGFR/PI3K/AKT pathway and inhibited cell migration by reducing the expression levels of MMP-2 and ICAM-1 (82). Zhang et al. (83) modified the *P. grandiflorum* polysaccharide (PGP40-1) using the HNO3/Na2SeO3 method to obtain seleniumated *P. grandiflorum* polysaccharide (Se-PGP40-1). Se-PGP40-1 could inhibit tumor proliferation and migration by inducing cell apoptosis and blocking angiogenesis.

### 5.4 Inhibition of new vascular growth

The metastasis and recurrence of tumors are closely related to neovascularization, which is a complex physiological process and is considered a marker of cancer progression (74). The VEGF is a highly characteristic factor that promotes endothelial cell growth and has the effect of promoting angiogenesis. The VEGF and IL-8 play crucial roles in promoting tumor angiogenesis by directly enhancing processes like endothelial cell proliferation, adhesion, migration, and angiogenesis. Therefore, developing drugs with high selectivity for VEGF could improve the prognosis of patients with metastatic cancer. The anti-angiogenic effects of platycodin D have recently been studied. Heydarzadeh et al. (84) have shown that platycodin D inhibited HUVEC proliferation, migration, and angiogenesis in a dose-dependent manner. The anti-angiogenic activity can be achieved by inhibiting VEGF induced p-VEGFR-2 and downstream targets. In studies of non-small cell lung cancer, platycodin D has been shown to hinder A549 cell proliferation and trigger apoptosis by modulating the p53/VEGF/MMP2 pathway (85). Meanwhile, in the study of esophageal cancer, apigenin was known as a tumor cell IL-6 transcription inhibitor, which inhibited the transcription and expression of IL-6, thereby inhibiting Eca-109 and Kyse-30 cell proliferation, inducing angiogenesis, and promoting apoptosis of esophageal cancer cell (86).

### 5.5 Regulating cell autophagy

Autophagy, known as type II programmed cell death, plays a dual role in the development of cancer. First, autophagy can maintain genomic stability and prevent the occurrence, proliferation, invasion, and metastasis of tumors. In addition, autophagy can also serve as a cellular defense mechanism, maintaining functional mitochondria, reducing DNA damage, and thus maintaining tumor development, and even promoting tumor tissue metastasis and drug resistance (87). Research has shown that platycodin D can trigger autophagy in different cancer cells (A549, H358, MCF7, HT29, and HepG2) (88, 89). In non-small cell lung cancer, Zhao et al. (90) found that *P. grandiflorum* induced autophagy in NCI-H460 and A549 cells by inhibiting the PI3K/AKT/mTOR signaling pathway and activating the JNK/MAPK signaling pathway. In liver cancer, platycodin D has anti-tumor effects of inhibiting BEL-7402 cell proliferation and inducing cell apoptosis. At the same time, it can trigger autophagy by activating the ERK and JNK pathways (91). In liver cancer research, luteolin can reduce the activity of SMMC-7721 cells, induce apoptosis, upregulate Caspase-8, downregulate the expression level of Bcl-2, and promote the transformation of LC-I to LC3-II, increase the expression of Beclin-1, and activate autophagy (92). In gastric cancer research, apigenin inhibited APG induced autophagy through endoplasmic reticulum stress and inhibited HIF-1α inducing endoplasmic reticulum stress and autophagy with Ezh2 to activate AGS tumor cell death (93).

**Figure 3:**
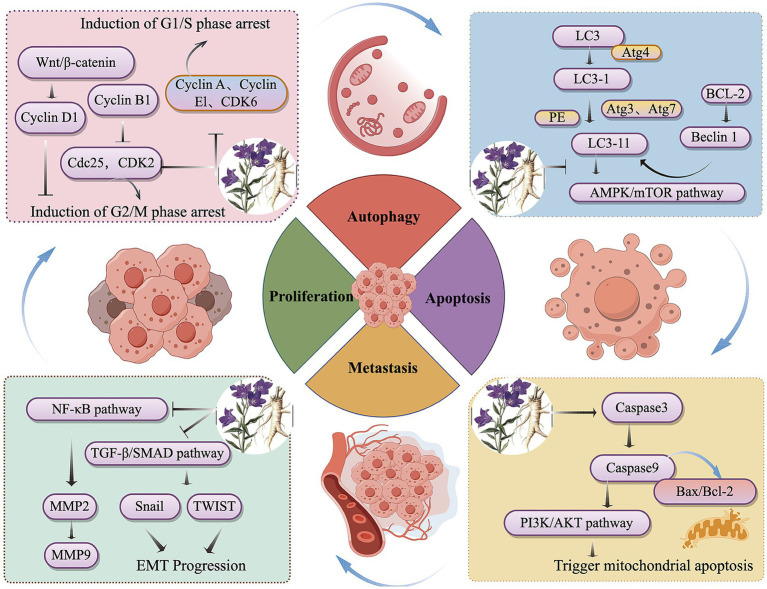
Mechanism of the active of ingredients in *P. grandiflorus* anti-tumors (by Figdraw, http://www.figdraw.com/).

**Figure 4:**
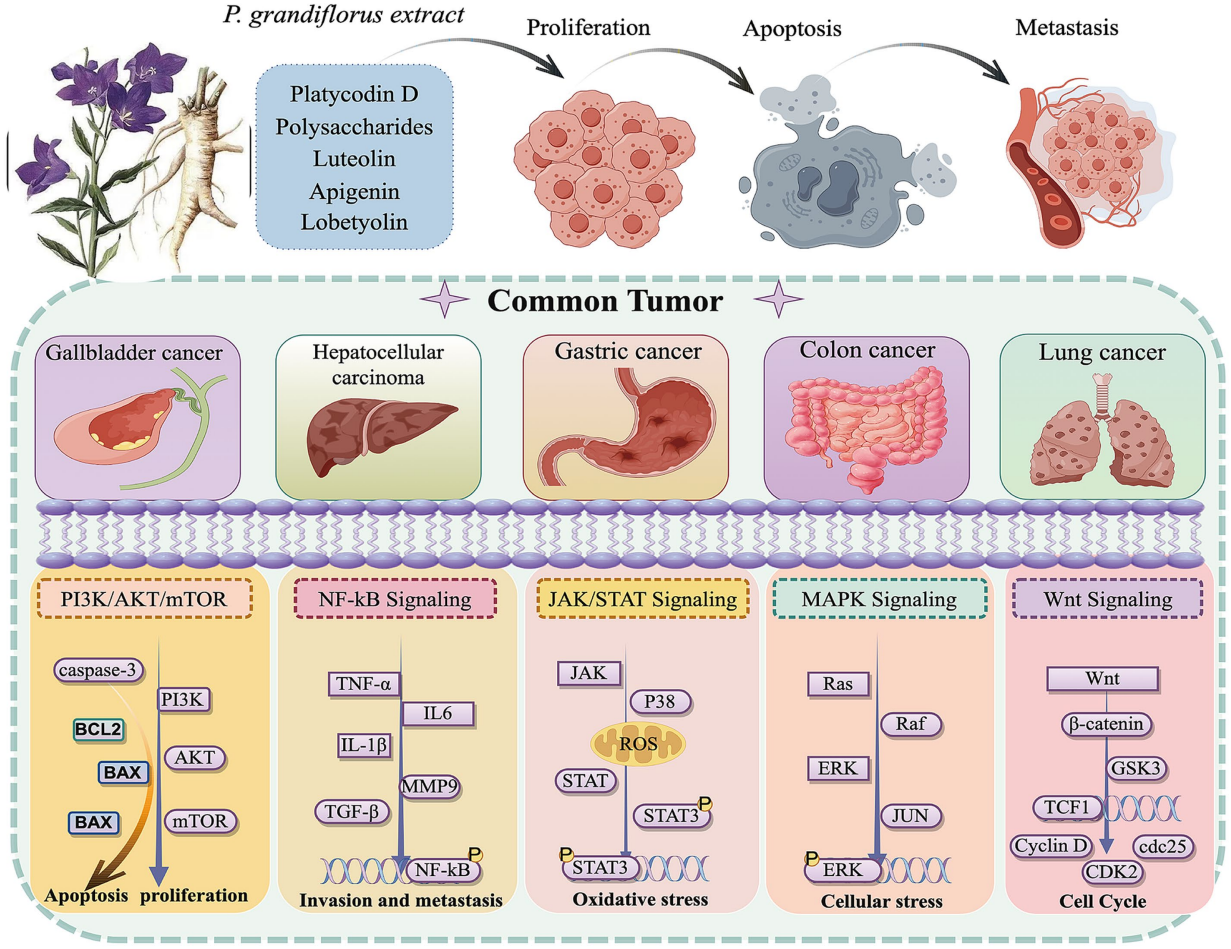
Ṭhe extract of *P. grandiflorus* inhibits cancers (such as Gallbladder cancer, Hepatocellular cancer, Gastric cancer, Lung cancer) through multiple signaling pathways (by Figdraw, http://www.figdraw.com/).

In summary, this study found that *P. grandiflorum* can inhibit tumor progression through multiple components, multiple targets, and multiple pathways. Based on literature review, among its active components, platycodin D has been the most extensively studied for its anti-tumor effects, with flavonoids (such as apigenin and luteolin) and polysaccharides also playing important roles. Its inhibitory effects have been demonstrated in various tumor cells (e.g., lung cancer cells, hepatoma cells), primarily through the PI3K/AKT signaling pathways. In addition, *P. grandiflorum* exerts its anti-tumor effects by inhibiting tumor cell proliferation, promoting apoptosis, and preventing metastasis, etc. The anticancer signaling pathways of *P. grandiflorum* extract are illustrated in Figure 3. Furthermore, given the diverse molecular mechanisms underlying cancer, the mechanisms involved in the tumor-inhibiting process of *P. grandiflorum* extracts and their active components are shown in Figure 4.

## 6 The effect-enhancing and toxicity-reducing of the active ingredient derived from *P. grandiflorum*

### 6.1 The protective effects on the liver and kidneys

**Figure 5:**
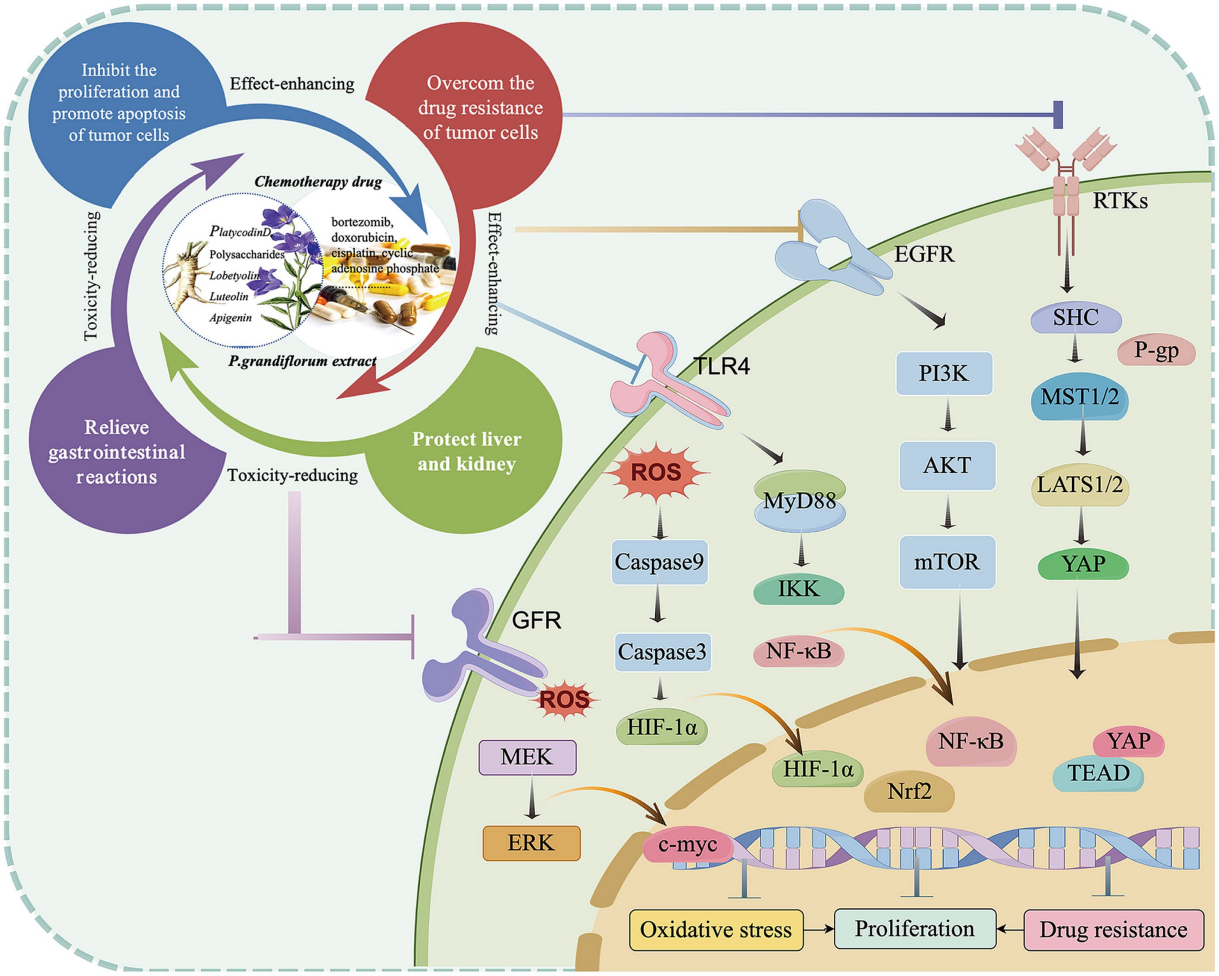
The effect-enhancing and toxicity-reducing molecular mechanism of the active ingredient derived from *P. grandiflorus* (by Figdraw, http://www.figdraw.com/).

Some active ingredients in *P. grandiflorum* not only have anti-cancer effects, but also have functions such as protecting the liver and reducing nephrotoxicity. Kim et al. (94) found that the fermentation extract of *P. grandiflorum* can alleviate acute liver injury induced by endotoxin in mice through reducing liver cell apoptosis and oxidative stress. Platycodin D protected against liver damage from acetaminophen by inhibiting oxidative stress and cell apoptosis. In addition, platycodin D can inhibit cisplatin-induced cell damage through various pathways, reverse cell apoptosis induced by Bax, Bad, Caspase-3, Caspase-9, and modulate PI3K/Akt and ERK/JNK/p38 signaling pathways, providing evidence for reduced cisplatin-induced nephrotoxicity. Apigenin has antioxidant, liver protective, anti-viral, and anti-inflammatory effects. In the treatment of a rat model of liver injury induced by chemotherapy drug cyclophosphamide, it can reduce liver function indicators, prevent liver cell damage, and inhibit ROS, LPO, NF- *κ* B. Pro-inflammatory mediators and indicators of apoptosis by activating Nrf2 signaling and reducing oxidative damage and inflammatory responses (95). Similarly, luteolin can also have a protective effect on liver cell damage caused by acetaminophen (96). *P. grandiflorum* polysaccharides also have certain liver-protective activity. Hou et al. (97) prepared nano-selenium saikosaponin complexes using the vitamin C reduction method. They administered the drugs by gavage and compared the protective effects of the nano-selenium saikosaponin complex and saikosaponin on liver-damaged mice. In addition, Liu et al. (98) found that the *P. grandiflorum* polysaccharides also have anti-inflammatory and antioxidant effects, and can regulate the immune function of the colon. They can alleviate ulcerative colitis caused by DSS through the mesenteric lymph circulation. Hou et al. (99) found that *Lobetyolin*, a Q-marker isolated from radix of *P. grandiflorum*, exerted protective effects on cisplatin-induced cytotoxicity in HEK293 cells (Figure 5; Table 4).

**Table 4:** Toxic reducing active ingredients and mechanism of *P. grandiflorum.*

Ingredient	Disease	Experimental model	Mechanism	Phenotype	References
Apigenin	Alleviated liver injury	A rat model of liver injury induced by cyclophosphamide	Inhibited ROS, MDA, NO, NF- κ B p65, iNOS, inflammatory factors, oxidative DNA damage, Bax and caspase-3 in cyclophosphamide-intoxicated rats; API enhanced liver antioxidants and Bcl-2, increased the expression of mRNA and protein of Nrf2 and HO-1.	Oxidative stress, apoptosis, inflammation response	(95)
Luteolin	Alleviated liver injury	L02 human liver cell injury model induced by acetaminophen	Reduced cell apoptosis and MDA content; Improved the activity of GSH and SOD; Upregulation of Bcl-2 and downregulation of Bax, caspase-3 mRNA expression.	Oxidative stress Apoptosis	(96)
Platycodin D	Alleviated liver injury	Male ICR mice	Reduced oxidative stress and inflammatory responses on alcohol-induced oxidative liver damage.	Oxidative stress and inflammatory response	(149)
	Induced nephrotoxicity	HEK-293	Inhibited cisplatin-induced cytotoxicity by inhibiting ROS-mediated oxidative damage, apoptosis, and inflammation.	Oxidative stress, apoptosis, inflammatory response	(150)
	Alleviated acute lung injury	MLE-12	Reduced the expression of TNF-*α*, IL-6, NF- κ B, Caspase-3, and Bax; Increased the expression level of Bcl-2.	Apoptosis and inflammation response	(151)
*P. grandiflorum* polysaccharides	Liver-protective activity	CCl4-induced mice liver injury model	Inhibited lipid peroxidation reactions, reduced the release of inflammatory factors, and regulated the expression of endoplasmic reticulum stress-related proteins (such as NLRP3)	Oxidative stress, inflammation response	(152)
Lobetyolin	Alleviated nephrotoxicity	HEK293 cells	Reduced the expression level of TNF-α, p-NF-κB, and IκBα in HEK293 cells	Inflammation response	(99)
	Alleviated liver injury	LPS-induced mice sepsis model	Reduce the levels of pro-inflammatory factors (TNF-α, IL-6, and IL-1β) in the sepsis mouse model induced by LPS	Inflammation response	(153)

In summary, the primary active components of *P. grandiflorum* listed in the table—including platycodin D, flavonoids, polysaccharides, and lobetyolin—have demonstrated significant liver and kidney protective effects in both *in vitro* cell experiments and animal models. Their key mechanism involves regulating key signaling pathways such as PI3K/AKT, ERK/JNK, and NF-κB, thereby inhibiting oxidative stress responses and inflammatory cascades, which contributes to their organ-protective effects. The emphasis of these components is different: platycodin D exhibits liver and kidney protective effects in vitro and *in vivo*, and also protects against acute lung injury; flavonoids show notable protective effects against acute liver injury in animal experiments; polysaccharides have confirmed liver protective effects in animal studies; and lobetyolin demonstrates liver and kidney protective potential in both in vitro and in vivo models. However, current research is limited to in vitro and animal studies, with no published clinical data available. Due to the absence of human pharmacokinetic information (such as drug concentration distribution in blood and target tissues), clinical efficacy indicators (such as symptom improvement and pathological changes), and safety evaluations (including adverse event incidence and dose tolerance), it is currently impossible to accurately assess the actual efficacy and potential risks of these components in humans. Therefore, their clinical translational value requires further validation through well-designed, standardized clinical trials.

### 6.2 Combined use of chemotherapeutic drugs

Chinese medicines can significantly enhance sensitivity to chemotherapeutic drugs, enhance tumor suppression and inhibit adverse reactions to chemotherapeutic drugs (100). Currently, there are more studies on Chinese medicine monomers and their active ingredients, and the combination of Chinese medicine with anti-tumor chemotherapeutic drug treatment can achieve significant tumor inhibition, reduce drug resistance, and improve adverse reactions and patients’ quality of life (101). Natural compounds frequently exhibit more diverse “Drug like” and “Biological friendliness” characteristics than pure synthetic compounds, and have enormous potential in future drug research and development. The literature consistently demonstrates that the most valuable aspect of the main active ingredients of *P. grandiflorum* lies in its ability to exert anti-tumor activity and synergistic effects with various anticancer chemotherapy drugs, while virtually having no cytotoxic effects on normal cells, and also having excellent auxiliary properties. *P. grandiflorum* has low toxicity and side effects, which is a traditional Chinese herbal medicine with similar medicinal and nutritional uses and has great potential for development. For example, platycodin D is widely used in combination therapy and adjuvant therapy to enhance anticancer and adjuvant activity. Huang et al. (102) found that the synergistic effect of platycodin D and sorafenib inhibited p-AKT, thereby promoting sorafenib induced cell apoptosis and G0/G1 phase cell cycle arrest in the PC3 cell line. In multiple myeloma cell lines, Wu et al. (103) demonstrated that platycodin D and bortezomib (BTZ) also exhibited this synergistic effect, significantly increasing the apoptosis of NCI-H929 and U266B1 cells. In addition, Tan et al. (104) found platycodin D and doxorubicin (DOX) worked synergistically to increase cellular uptake of doxorubicin, enhance its inhibitory effect on cell proliferation, and inhibit DOX anti-inflammatory effect in MCF-7 and MDA-MB-231 cells. Lei et al. (105) discovered that platycodin D, without damaging the membranes of organelles, can promote the escape within the body, thereby reversing the drug resistance of HepG2 cells to chemotherapy drugs. Zhang et al. (106) observed that the aqueous extract of *P. grandifloras* and platycodin D can affect the Cyclophosphamide (CTX) pharmacokinetic parameters (delays the absorption process of CTX in the body, reduces the peak concentration, and makes the absorption process more gradual). The combination of *P. grandiflorum* extract or platycodin D has a reducing-toxicity and enhancing-effect significance on CTX. To sum up, these results indicates that platycodin D may enhance efficacy and reduce toxicity by reversing drug resistance and influencing the pharmacokinetics of chemotherapeutic drugs.

In addition to platycodin D, the combination of some flavonoids in *P. grandiflorum* with doxorubicin can also alter the pharmacokinetics of doxorubicin *in vivo*. For instance, the combination of apigenin and doxorubicin can hinder the growth of HepG2 cells, increase the G0/G1 phase arrest of liver cancer cells, and increase the apoptosis of hepatocellular carcinoma (107). Similarly, Ahmed et al. (108) found that the combined of luteolin and doxorubicin can enhance the cytotoxicity of breast cancer, inhibit its proliferation and promote apoptosis. Chen et al. (109) found that luteolin increased the sensitivity of cisplatin induced apoptosis in HepG2 cells by upregulating p-JNK protein expression. Overall, studies have shown that the combination of the active ingredients of *P. grandiflorum* and anti-tumor chemotherapy drugs (bortezomib, doxorubicin, cisplatin, cyclic adenosine phosphate) performed adequately in promoting the uptake of therapeutic drugs, promoting therapeutic efficacy, and reducing the damage of chemotherapy drugs to normal cells. Therefore, the active ingredients of *P. grandiflorum* can be used as a synergistic herbal medicine for treating tumors and has broad clinical application potential.

## 7 New drug delivery systems anti-tumor-nanotechnology

The natural extracts of *P. grandiflorum* (such as platycodin D, flavonoids, and polysaccharides) exhibit significant antitumor activity. However, these active components generally suffer from low water solubility, and the amount that enters the bloodstream via traditional administration routes is insufficient—this not only impairs their oral bioavailability but also restricts their clinical application (110). Therefore, improving the bioavailability of the active components from *P. grandiflorum* is crucial for the prevention and treatment of cancer. Studies have shown that nanodelivery systems can significantly enhance therapeutic efficacy by prolonging drug circulation time, reducing side effects, improving bioavailability, and achieving tissue targeting (111). In recently years, the widely studied nanoparticulate drug delivery systems mainly include solid lipid nanoparticles, liposomes, chitosan nanoparticles, polymeric micelles, hydrogels, etc. (112). The differences in their key properties are determined by carrier materials, structural design, and functional positioning.

Liposomes research is the most extensive, which exhibit advantages such as low toxicity, high targeting ability, high biodegradability, and ease of functionalization. These properties enable them to facilitate the targeted delivery of the drugs they carry, thereby exerting unique advantages in tumor therapy (113). Among them, Solid lipid nanoparticles (SLNs) have a wide range of applications in drug delivery systems, which can be used to encapsulate drugs and achieve targeted delivery to improve drug efficacy and reduce adverse reactions (114). Polymer nanoparticles are mainly used for the surface modification of small molecules like RNA and proteins, with the aim of enhancing the targeting ability of antitumor drugs (115). Hydrogels are composed of hydrophilic polymers and are similar to the extracellular matrix, possessing good biocompatibility. They can be modified through processes that prevent the degradation of biomolecules during delivery while maintaining the sustained release of drugs (116). Chitosan nanoparticles have good biocompatibility and safety. They are suitable for oral administration, capable of overcoming the intestinal barrier, and exhibit sustained and controlled release properties. Furthermore, they have no restrictions on gene size and are simple to prepare (117). Currently, Conjugated nanodelivery systems, such as polymer-lipid hybrid nanoparticles (PLHNPs), not only have strong stability and high drug-loading capacity but also possess long circulation time and excellent biodegradability (118). In addition to the aforementioned organic nanoparticles, inorganic nanoparticles (such as silica, iron oxide, gold nanoparticles, and selenium nanoparticles, etc.) have attracted considerable attention in cancer treatment. They can be used both as carriers for drug delivery and as active pharmaceutical ingredients themselves, exhibiting great potential and safety in the field of medical applications (119).

**Figure 6:**
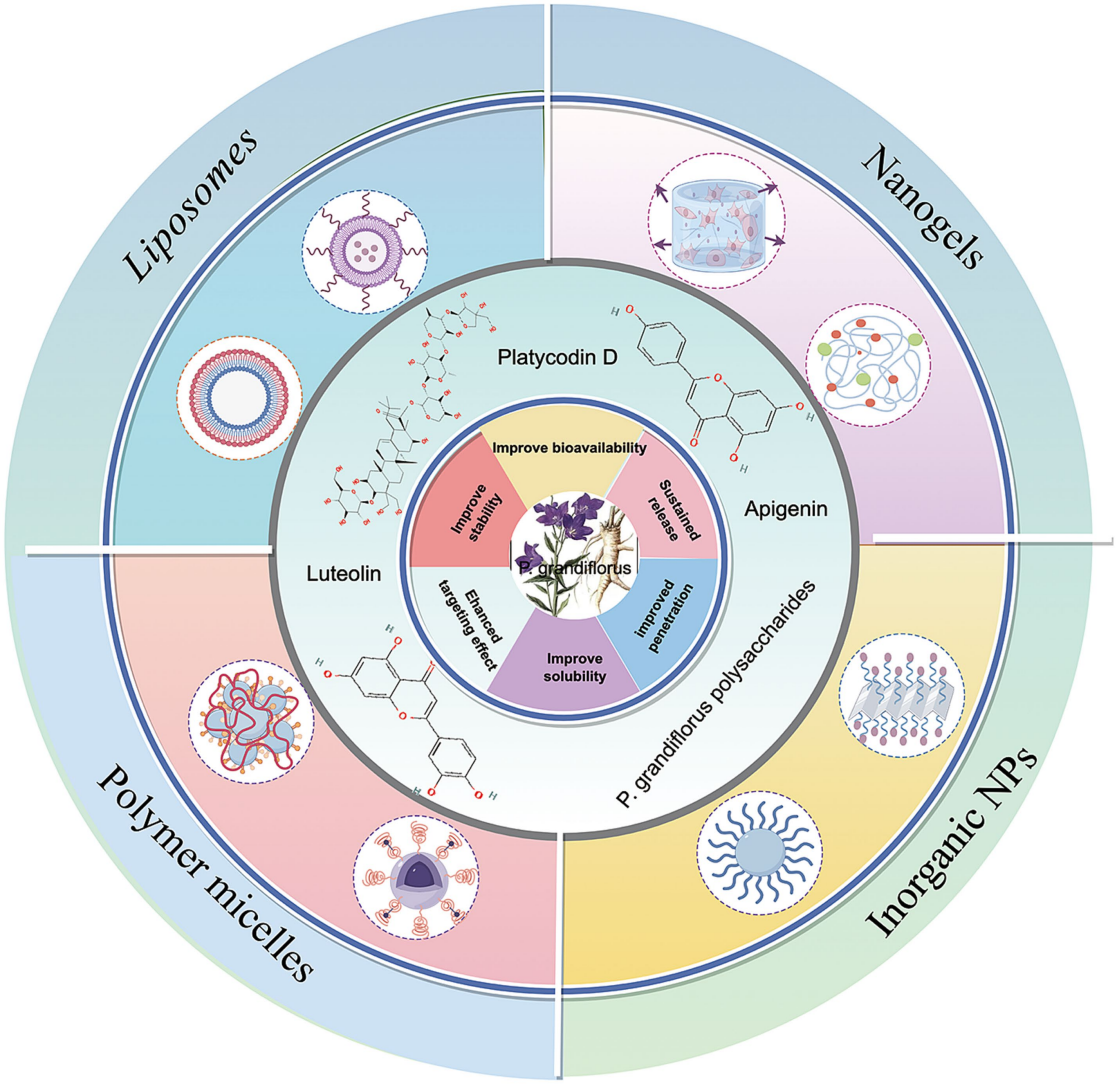
Application of *P. grandiflorus* extract in nanoparticle drug delivery system (by Figdraw, http://www.figdraw.com/).

Notably, the new drug delivery system has significantly improved the anti-tumor activity of *P. grandiflorum* extract. Consequently, the integration of *P. grandiflorum* extract with nanotechnology held considerable promise for the future development of clinical anti-tumor drugs (as shown in Figure 6). Firstly, the combination of platycodin D and liposomes nanoparticles becomes an effective drug delivery method and effectively improves drug stability, bioavailability, targeting of platycodin D and prolong its circulating time *in vivo*. Kim et al. (120) demonstrated that the combination of platycodin D and liposome nanotechnology enables the delivery of therapeutic cargoes to cells, offers enhanced protection for drugs during the delivery process, and exhibits no hepatotoxicity. In colorectal cancer, we developed platycodin D2 and liposomes as a tumor-targeting therapy. The platycodin D2-liposomes treatment demonstrated a successful tumor-targeting ability in the colorectal cancer xenografts, in which PCD2 not only exerted a potent antitumor effect by inducing apoptotic cell death and but also functioned as a liposome membrane stabilizer (121). Luteolin, as one of the flavonoids in *P. grandiflorum*. Cao et al. (122) developed luteolin-loaded nanoliposomes that target the PG-1 receptor on the surface of liver cancer cells, enhancing drug accumulation at the tumor site. Meanwhile, these nanoliposomes increase the expression of lactate dehydrogenase (LDH), disrupting tumor growth pathways and thereby addressing the compound’s low oral bioavailability. Adel et al. (123) found that apigenin bound liposomes enhanced the bioavailability and chemical stability of apigenin in breast cancer, thereby enhancing the anti-breast cancer effect. In the xenograft tumor models of human colorectal cancer HCT-15 and HT-2 cells (124), Apigenin liposomes not only enhance the apoptosis-inducing effect on tumor cells, but also improve the hemocompatibility and cytocompatibility with normal fibroblasts, thereby reducing drug toxicity. In addition to liposome nanoparticles, chitosan nanoparticles (ChNPs) can also introduce chemotherapy drugs into cells, increase their activity, and release encapsulated compounds over time (125). In liver cancer, Mabrouk et al. (126) first added apigenin to chitosan to increase its hydrophobicity, and then coated the chitosan surface with folate albumin to increase its stability and bioavailability. Shi et al. (127) found an oral herbal system-nanoparticle mixture (HNS), which is formed through self-association of *P. grandiflorum*-*Curcuma zedoaria*, and combined with lipid-polymer nanoparticles (LPNs). HNS induced cell apoptosis and regulated the expression of MMP-9 and TGF-β1, thereby reducing lung metastasis in 4 T1 tumor-bearing mice and altering the tumor microenvironment. In addition, more and more evidence indicated (128) that *P. grandiflorum* polysaccharides can be used as a stabilizer to modify nano-selenium particles, thereby enhancing their stability in solution. The polysaccharide PGP-AE can inhibit gastric cancer cells *in vitro*, but its anti-tumor effect *in vivo* is not significant; while PGP90 needs to be combined with nano-selenium to significantly enhance its activity, indicating that nano-modification is crucial for the efficacy of the drug.

*P. grandiflorum* extract combined with nanoparticles have been widely studied in cancer treatment. In conclusion, platycodin D mainly exerts its effects by combining with liposomes, while flavonoid components mostly enhance antitumor efficacy with the help of solid nanoparticles, liposomes, and chitosan nanocarriers. In addition, polymer-based nano-delivery systems are often combined with polysaccharide components, which can significantly improve anti-cancer activity. It can be seen that nanopharmaceutical delivery systems address issues such as low solubility and poor bioavailability of the active components from *P. grandiflorum*, significantly optimizing them in vivo pharmacokinetic and pharmacodynamic properties, and providing a feasible technical pathway for the clinical translation of these active components.

## 8 Homology of medicine and food: preventive healthcare

### 8.1 Clinical trials

In the past decade, researchers have discovered the new pharmacological potential of platycodin in the treatment of chronic conditions, including hyperlipidemia, hypertension, diabetes and obesity (129). *P. grandiflorum*, as a homologous plant of medicine and food, not only exhibits anti-tumor potential, which also plays many important roles in prevention and healthcare (15). We performed a search using the keywords “*Platycodon grandiflorum*” and “preventive healthcare” to identify relevant clinical trials on the International Clinical Trials Registry Platform^10^ (accessed June 12, 2025). The search results indicated (130) that *P. grandiflorum* extract (GCWB107) can reduce blood lipids in patients with obesity (trial registration number: NCT04023864). Another study discovered (131) that *P. grandiflorum* extract can enhance human immune function (trial registration number: KCT0005945). In addition, *P. grandiflorum* extract mixture also can effectively improve oxidative stress (trial registration number: KCT0007161).

These preliminary trials indicate safety, but efficacy data are limited. In addition, *P. grandiflorum* oral medicine may have certain potential adverse reactions, with common ones including gastrointestinal discomfort and allergic reactions; high-dose use or long-term administration may also cause liver damage. In terms of drug-nutrient interactions, concurrent use of these preparations with anticoagulant drugs may increase the risk of bleeding, while co-administration with gastrointestinal drugs can affect the absorption of their own active ingredients, resulting in absorption disorders (132). Therefore, further clinical verification of the efficacy of *P. grandiflorum* is required. Only through clinical trials can the optimal dosage (suitable for specific populations) and administration method be determined, and its side effects and potential risks be comprehensively evaluated. This process ensures that the drug data are thoroughly validated and comply with research and regulatory standards.

### 8.2 Preventive healthcare product

*P. grandiflorum* is integrated into daily therapeutic or preventive medicine on the basis of its properties as both a drug and a food. This practice not only improves the edible va*lue of P. grandiflorum,* but also maximizes the medicinal value of *P. grandiflorum*. Currently, there are numerous health products containing *P. grandiflorum* as a primary ingredient in treatments and preventive medications available on the market. A search of the Special Food Information Query Platform^11^ (accessed June 22, 2025) of China’s State Administration for Market Regulation reveals that there are currently 11 health products with a “National Food Health Note” on the market. The *P. grandiflorum*-based health products are recognized not only for their ability to alleviate throat discomfort and enhance laryngeal health but also for their role in boosting immunity, as shown in Table 5. However, *P. grandiflorum* preparations still face challenges in regulation and dose standardization. Specifically, natural medicine complex composition—containing saponins, polysaccharides, flavonoids, and other active substances—further exacerbates difficulties in standardization; meanwhile, variations in raw materials (e.g., variety, origin) and dosage forms can cause fluctuations in the content and absorption efficiency of active ingredients, rendering precise dose control difficult (133). Therefore, to ensure the quality of health products, it is essential to emphasize clinical validation and strengthen regulatory oversight of products classified under “food-medicine homology.

**Table 5:** The main healthcare product of *P. grandiflorum.*

Efficacy	Product name	Component	Approval number	Signature ingredient content (100 g)
Relievie throat discomfort and improve laryngeal health; boost immunity	Chuanbei loquat cream (Sugar-free formula)	Loquat pulp, *Platycodon grandiflorum* (Jiegeng, *P. grandiflorum*), Fritillaria cirrhosa (Beimu), Glycyrrhiza uralensis (Gancao), Exocarpium citri rubrum, Menthol, Maltitol, Water.	G20130229	Total flavonoids 96.0 mg/100 g
	Yuganzi Jiegeng syrup	*Phyllanthus emblica*, *Platycodon grandiflorum* (*P. grandiflorum*), Glycyrrhiza uralensis (Gancao), Exocarpium citri rubrum, Scrophularia ningpoensis (Xuansheng), *Ophiopogon japonicus* (Maidong), Sucrose, Citric acid, Water.	G20100396	Total saponins 0.42 g/100 g; total flavonoids 340 mg/100 g
	Jinyinhua Chinese white olive Jiegeng extract lozenge	Honeysuckle (Jinyinhua) extract, Chinese white olive extract, *Platycodon grandiflorum* (*P. grandiflorum*) extract, Menthol.	G20140807	Chlorogenic acid 500 mg/100 g
	Jinyinhua Jiegeng Fig extract lozenge	Fig (irradiated), *Platycodon grandiflorum* (*P. grandiflorum*), Honeysuckle extract (Jinyinhua).	G20141269	Crude polysaccharide 4.34 g/100 g; chlorogenic acid 0.34 g/100 g
	Jiegeng Dihuang lozenge	Scrophularia ningpoensis (Xuansheng), Rehmannia glutinosa (Dihuang), *Ophiopogon japonicus* (Maidong), *Platycodon grandiflorum* (*P. grandiflorum*), Pericarpium Citri Reticulatae (irradiated).	G20150294	Total saponins 0.24 g/100 g
	Jiegeng Gancao lozenge	*Platycodon grandiflorum* (*P. grandiflorum*) extract, Glycyrrhiza uralensis (Gancao) extract, Siraitia grosvenorii extract, *Phyllanthus emblica* extract, *Prunus mume* extract, D-Mannitol, Lactose, Dextrin, Film coating agent.	G20240044	Total saponins 0.1 g/100 g; total flavonoids 40 mg/100 g

## 9 Summary and future prospects

Cancer is a complex malignancy, not only because of its diverse proliferation and metastasis, but also because cancer cells are highly adaptable. Chinese herbal medicines, as natural compounds, often have diverse chemical structures and biological activities, and are widely studied and applied in the prevention and treatment of multiple tumors. Through an analysis of relevant literature and network pharmacological results, this paper showed that the anti-tumor active ingredients of *P. grandiflorum* include platycodin D, *P. grandiflorum* polysaccharides, Lobetyolin, luteolin, and apigenin. In this paper, we investigated the mechanism of *P. grandiflorum* on cell proliferation, cell cycle, and cell migration based on its active components. It has been found that platycodin D mainly hindered cancer cell proliferation and induced cancer cell apoptosis, which is the main mechanism for producing anti-cancer activity. Apigenin mainly inhibited tumor growth, induced endogenous apoptosis and autophagy, and inhibited angiogenesis as the main mechanism for producing anti-cancer activity. Luteolin and Lobetyolin mainly inhibited the growth, migration and invasion of tumor cells, which were the main mechanisms of tumor activity. *P. grandiflorum* polysaccharides can stimulate the activity of immune cells and macrophages, enhance the functions of T cells and NK cells, thereby improving the body’s immune surveillance and clearance ability against tumors. Additionally, we explored the application of extracts from *P. grandiflorum* in modern drug development. These extracts can be combined with certain chemotherapy drugs to target tumor tissues based on their anti-tumor effects, ultimately enhancing efficacy while reducing toxicity. With the emergence of new drug delivery systems, the challenges of low utilization and poor solubility of active ingredients in traditional oral Chinese medicine have been extensively studied. Among them, Nano-delivery systems have the advantages of high bioavailability, strong targeting ability, and good sustained release effect, gradually improving the limitations of *P. grandiflorum* extract anti-tumor applications. Furthermore, as an herbal medicine, *P. grandiflorum* has a long history of medicinal and edible use. In recently years, *P. grandiflorum* also plays many important roles in prevention and health care of cancer. However, there is a lack of large-scale, rigorously designed, and well-implemented clinical trials to confirm the anti-cancer properties of *P. grandiflorum.* With the continued development of research methods and the deepening of the level of research, it is worth looking forward to the application of the active ingredients of *P. grandiflorum* in the development of clinical drug formulations with anti-tumor effects. Therefore, in the future, we need to strengthen a series of clinical application research based on sufficient basic research, expanding the scope of clinical application from conventional applications to various fields such as cancer, antiviral agents, vaccine adjuvants, etc.

## Footnotes

^1^ https://pubmed.ncbi.nlm.nih.gov

^2^ https://scholar.google.com

^3^ https://www.sciencedirect.com

^4^ https://www.webofscience.com

^5^ https://tcmsp-e.com/

^6^ https://www.uniprot.org/uniprot/

^7^ http://swisstargetprediction.ch/

^8^ https://metascape.org/

^9^ www.bioinformatics.com.cn

^10^ https://trialsearch.who.int/

^11^ http://ypzsx.gsxt.gov.cn/specialfood/#/food
